# Stress echo 2020: the international stress echo study in ischemic and non-ischemic heart disease

**DOI:** 10.1186/s12947-016-0092-1

**Published:** 2017-01-18

**Authors:** Eugenio Picano, Quirino Ciampi, Rodolfo Citro, Antonello D’Andrea, Maria Chiara Scali, Lauro Cortigiani, Iacopo Olivotto, Fabio Mori, Maurizio Galderisi, Marco Fabio Costantino, Lorenza Pratali, Giovanni Di Salvo, Eduardo Bossone, Francesco Ferrara, Luna Gargani, Fausto Rigo, Nicola Gaibazzi, Giuseppe Limongelli, Giuseppe Pacileo, Maria Grazia Andreassi, Bruno Pinamonti, Laura Massa, Marco A. R. Torres, Marcelo H. Miglioranza, Clarissa Borguezan Daros, José Luis de Castro e Silva Pretto, Branko Beleslin, Ana Djordjevic-Dikic, Albert Varga, Attila Palinkas, Gergely Agoston, Dario Gregori, Paolo Trambaiolo, Sergio Severino, Ayana Arystan, Marco Paterni, Clara Carpeggiani, Paolo Colonna

**Affiliations:** 1 Institute of Clinical Physiology, National Research Council, Pisa, Italy; 2 Cardiology Division, Fatebenefratelli Hospital, Benevento, Italy; 3 Heart Department, University Hospital “San Giovanni di Dio e Ruggi d’Aragona”, Salerno, Italy; 4 Division of Cardiology, Monaldi Hospital, Second University of Naples, Naples, Italy; 5 Cardiology Department, Pisa University and Nottola (Siena) Hospital, Pisa, Italy; 6 Cardiology Department, San Luca Hospital, Lucca, Italy; 7 Cardiology Department, Careggi Hospital, Florence, Italy; 8 Department of Advanced Biomedical Sciences, Federico II University Hospital, Naples, Italy; 9 Cardiology Department, San Carlo Hospital, Potenza, Italy; 10 Pediatric Cardiology Department, Brompton Hospital, London, UK; 11 Division of Cardiology, Ospedale dell’Angelo Mestre-Venice, Mestre, Italy; 12 Cardiology Department, Parma University Hospital, Parma, Italy; 13 Pediatric Cardiology Department, Monaldi Hospital Clinics, Naples, Italy; 14 Cardiology Department, University Hospital “Ospedale Riuniti”, Trieste, Italy; 15 Hospital de Clinicas de Porto Alegre, Universidade Federal do Rio Grande do Sul, Porto Alegre, Brazil; 16 Cardiology Institute of Rio Grande do Sul, Porto Alegre, Brazil; 17 Cardiology Division, Hospital San José, Criciuma, Brazil; 18 Hospital Sao Vicente de Paulo, Hospital de Cidade, Passo Fundo, Brazil; 19 Cardiology Clinic, Clinical Center of Serbia, Medical School, University of Belgrade, Belgrade, Serbia; 20 Institute of Family Medicine, University of Szeged, Szeged, Hungary; 21 Department of Internal Medicine, Elisabeth Hospital, Hodmezovasarhely, Hungary; 22 Department of Biostatistics, University of Padua, Padua, Italy; 23 Department of Cardiology, Sandro Pertini Hospital, Rome, Italy; 24 Cardiology Department, Monaldi Hospital, Naples, Italy; 25 RSE, Medical Centre Hospital of the President’s Affairs Administration of the Republic of Kazakhstan, Astana, Kazakhstan; 26 Cardiology Hospital, Policlinico of Bari, Bari, Italy

**Keywords:** Effectiveness, Imaging, Prognosis, Stress echocardiography

## Abstract

**Background:**

Stress echocardiography (SE) has an established role in evidence-based guidelines, but recently its breadth and variety of applications have extended well beyond coronary artery disease (CAD). We lack a prospective research study of SE applications, in and beyond CAD, also considering a variety of signs in addition to regional wall motion abnormalities.

**Methods:**

In a prospective, multicenter, international, observational study design, > 100 certified high-volume SE labs (initially from Italy, Brazil, Hungary, and Serbia) will be networked with an organized system of clinical, laboratory and imaging data collection at the time of physical or pharmacological SE, with structured follow-up information. The study is endorsed by the Italian Society of Cardiovascular Echography and organized in 10 subprojects focusing on: contractile reserve for prediction of cardiac resynchronization or medical therapy response; stress B-lines in heart failure; hypertrophic cardiomyopathy; heart failure with preserved ejection fraction; mitral regurgitation after either transcatheter or surgical aortic valve replacement; outdoor SE in extreme physiology; right ventricular contractile reserve in repaired Tetralogy of Fallot; suspected or initial pulmonary arterial hypertension; coronary flow velocity, left ventricular elastance reserve and B-lines in known or suspected CAD; identification of subclinical familial disease in genotype-positive, phenotype- negative healthy relatives of inherited disease (such as hypertrophic cardiomyopathy).

**Results:**

We expect to recruit about 10,000 patients over a 5-year period (2016-2020), with sample sizes ranging from 5,000 for coronary flow velocity/ left ventricular elastance/ B-lines in CAD to around 250 for hypertrophic cardiomyopathy or repaired Tetralogy of Fallot. This data-base will allow to investigate technical questions such as feasibility and reproducibility of various SE parameters and to assess their prognostic value in different clinical scenarios.

**Conclusions:**

The study will create the cultural, informatic and scientific infrastructure connecting high-volume, accredited SE labs, sharing common criteria of indication, execution, reporting and image storage of SE to obtain original safety, feasibility, and outcome data in evidence-poor diagnostic fields, also outside the established core application of SE in CAD based on regional wall motion abnormalities. The study will standardize procedures, validate emerging signs, and integrate the new information with established knowledge, helping to build a next-generation SE lab without inner walls.

## Background

**Fig. 1:**
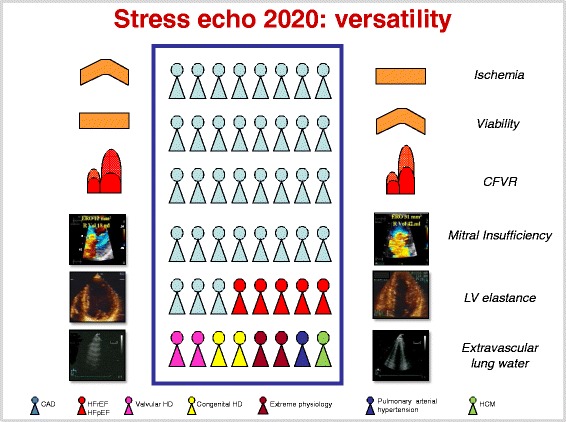
In the box, the contemporary spectrum of patients for whom SE can offer potentially unique diagnostic information: coronary artery disease; heart failure (with either reduced or preserved left ventricular function); hypertrophic cardiomyopathy; valvular heart disease; extreme physiology; adult repaired congenital heart disease; early, at risk, or borderline pulmonary arterial hypertension. For each clinical condition, a different key SE parameter can be used, evaluated at rest (*left* column) and during stress (*right* column), maximizing the versatility of the technique. From top to bottom rows, regional wall motion (for ischemia and viability), coronary flow velocity reserve (CFVR), mitral insufficiency, end-systolic volume of the left ventricle (necessary to assess left ventricular elastance), and B-lines (a marker of extravascular lung water). Modified and adapted from ref 4 (Picano and Pellikka [4])

For a long time, the scope of stress echo (SE) remained focused on coronary artery disease (CAD) [1, 2]. In the last ten years, SE has exploded in its breadth and variety of applications [3, 4]. From a one-fits-all approach (wall motion by 2D-echo in the patient with known or suspected CAD), the field has progressed to an omnivorous, next-generation laboratory employing a variety of technologies (from M-Mode to 2D, from pulsed, continuous, color and tissue Doppler to lung ultrasound) on patients covering the entire spectrum of severity (from elite athletes to patients with end-stage heart failure) and ages (from children with congenital heart disease to the elderly with aortic stenosis) [4] (Fig. 1). As a consequence of this rapid growth, the clinical use of SE often lacks the necessary supportive evidence and is slowed by unavoidable confusion on methodological issues in a rapidly evolving field. This situation represents a challenge and an opportunity for the SE community. It is a challenge, because in other fields of cardiology we currently lack the level of evidence collected in the last 30 years that led SE based on the detection of regional wall motion abnormalities to play a central role in CAD and heart failure management, as acknowledged in specialty [5, 6] and general cardiology guidelines [7–10]. It is also an opportunity, because today SE has the unprecedented advantages of economic sustainability, lack of radiation, portability and versatility making it especially attractive in the current era of increasing societal concerns about cardiac imaging costs and long-term risks due to ionizing radiation [11].

“Stress echo 2020” (SE2020) is a prospective, multicenter, international, observational study, involving > 100 SE laboratories, with short-term, mid-term, and long-term aims: 1) In the short term (12 months), to create the cultural, digital and scientific infrastructure connecting high volume, accredited SE labs, sharing common criteria of indication, performance, reporting and image storage of SE; 2) In the mid-term (2 to 3 years), to obtain original safety, feasibility, and outcome data in evidence-poor diagnostic fields, also outside the established core application of SE in CAD [12]: contractile reserve for prediction of cardiac resynchronization or medical therapy response; stress B-lines in heart failure; hypertrophic cardiomyopathy; heart failure with preserved ejection fraction; mitral and aortic valve function after transcatheter or surgical aortic valve implantation; outdoor SE in extreme physiology; right ventricular contractile reserve in repaired Tetralogy of Fallot; exercise-induced pulmonary artery pressure rise in predicting outcome in at risk, borderline or early established pulmonary arterial hypertension; added value of new, second- and third-generation parameters (coronary flow velocity reserve, left ventricular elastance reserve and B-lines) for refining prognosis based on regional wall motion abnormalities, within and outside CAD; identification of subclinical familial disease in genotype-positive and phenotype–negative healthy relatives at risk for hypertrophic cardiomyopathy or familial dilated cardiomyopathy or pulmonary hypertension; 3) In the long-term (at the end of the 5-year project), to establish an up and running platform for future prospective, randomized, outcome studies with selective interventions on specific diseases based on SE results.

## Methods

In a prospective, multicenter, international, observational study design, > 100 SE labs will be networked with systematic clinical, laboratory and imaging data collection at the time of SE and with structured follow-up information.

The study theater is the international network of cardiology SE laboratories, and the study is endorsed and promoted by the Italian Society of Cardiovascular Echography. The main documents (from protocols to case report form to software and website platform) will be in English, so that selected highly motivated and experienced centers may join specific subprojects, setting the stage for an international upscaling of the project in the coming years. The starting point of the recruitment phase was a recent electronic survey by the Italian Society of Cardiovascular Echography, in 2015 censoring 134 laboratories with moderate- (>100/year) to high- (>400/year) volume SE activities [13], which were precisely interrogated for interest in participation to SE2020. Laboratories from Brazil, Serbia, and Hungary have already joined the project. The recruitment plan forecasts 500 patients by the end of 2016, with doubling of the rate of enrollment in subsequent years, in parallel with the increasing number of recruiting labs fulfilling quality control criteria, reaching the target number of 100 at the end of the 5-year schedule.

### Data collection

As recommended by guidelines, we will adopt a 17-segment model of the left ventricle, with 1-to-4 segmental scoring system [5, 6]. Stress protocols are harmonized according to recent European and North-American scientific societies' guidelines, with semi-supine exercise recommended and pharmacological stress dosages up to 40 mcg/kg/min for dobutamine, up to 0.84 mg/kg in 6 min for dipyridamole, and up to a 4-min step of 200 microg/kg/min for adenosine [5, 6]. With dobutamine, atropine (up to 1 mg) can be administered in patients with suspected CAD (protocol 9), and it is associated with a higher rate of complications in those with a history of neuropsychiatric symptoms, reduced left ventricular function, or small body habitus. The maximal allowed dobutamine dose is 20 mcg/kg/min in patients with aortic stenosis, in whom higher doses are less safe and probably unnecessary [13]. All laboratories will share a standardized case report form coded in a database format to facilitate retrieval and communication. For applications outside CAD and for CAD testing with vasodilator stress, no atropine is given on top of pharmacological stress.

**Fig. 2:**
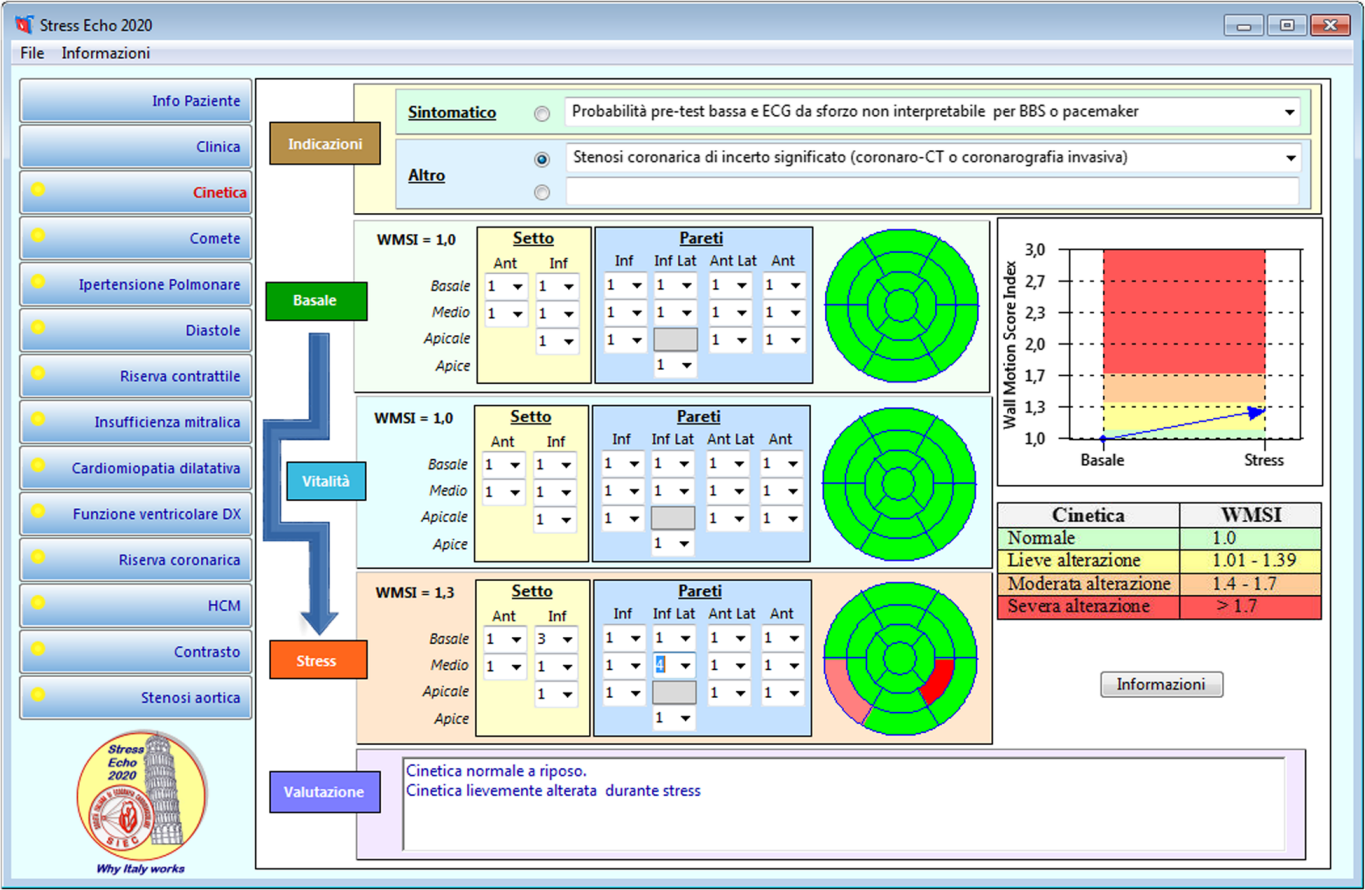
The computerized case report form for the regional wall motion abnormalities of the SE 2020 study. The grading of the response is reported in tabular (*right side*, *lower panel*) and graphic (*right side*, *upper panel*) format, with normal values of Wall Motion Score Index in *green*, mild impairment in *yellow*, moderate in *orange*, and severe impairment in *red*

Although data collection with a dedicated project-specific case report form is allowed, we encourage implementing a dedicated, free ad-hoc system for data storage and reporting developed at the National Research Council, Institute of Clinical Physiology. The software provides a suitable informatics infrastructure for the SE 2020 Italian multicenter study, with an intuitive graphic interface, eye-catching graphic format and convenient reporting option. It could represent the trade-off between the comprehensive information required by scientific standards and the smooth workflow priority of busy, high-volume, clinically-driven activities [14]. As an illustrative example, the report page for regional wall motion abnormalities and Wall Motion Score Index is shown in Fig. 2. The software was developed and tested in Italian and the translation of the last release in other languages (English, Portuguese and Serbian) is currently in progress.

### Data analysis

Data will be expressed as mean ± standard deviation (normally distributed data, such as wall motion score index), median and inter-quartile (25th, 75th) range (non-normally distributed data, such as B-lines) or per cent frequency (categorical data, such as presence or absence of severe mitral regurgitation), with absolute numbers. In patients with coronary angiographic information, diagnostic sensitivity, specificity, positive and negative predictive value will be assessed for any combination of the wall motion score index, coronary flow velocity reserve, left ventricular contractile reserve and B-lines.

One-sample comparisons will be performed using Wilcoxon test, and the chi-squared test without Fisher's correction for categorical data. Event rates will be estimated with Kaplan–Meier curves and compared by the log-rank test. Univariable analyses by Cox proportional hazards models will be performed to assess the association between each candidate variable and outcome. All variables with *P <*0.20 by univariable analysis will be considered as candidate variables for the multivariable analyses. Goodness of fit of the models will be based on C-statistics and its variants, adjusting for optimism using bootstrap replications (at least 1000). A receiver operating characteristic analysis will be used to obtain the best prognostic predictor for the individual SE variables. We will also analyze the data according to a clinically guided stepwise procedure, where the variables will be included in the model in the same order in which they are actually considered by the cardiologist. Statistical significance will be set at *p* < 0.05.

### Quality control

It is well-known that the diagnostic performance of SE is closely related to the level of expertise of the cardiologist-echocardiographer performing the test, since the evaluation of regional wall motion is subjective and qualitative, with considerable variability even among experienced centers of undisputed reputation [15]. The reproducibility and accuracy of wall motion reading can be substantially increased with limited training [16] and through development of conservative, pre-specified reading criteria [17]. Therefore, quality control of the diagnostic performance in the various laboratories is a must in order to enter meaningful information in the data bank. The burden of quality control is on the hub center of the principal investigator of each subproject, where various spoke centers may converge. For the general project, the hub center for regional wall motion analysis is Pisa-CNR, in coordination with the principal investigator. There are five different levels of quality control, with increasing levels of complexity:

1. Level 1, pre-requisite: a volume activity of the lab of at least 100 SE tests per year, which is the requirement for credentialing of SE activity by scientific societies [18].
2. Level 2, spoke centers read hub SE images, consisting in 20 selected studies for regional wall motion analysis. The concordance requires identification of test negativity/positivity and, in positive tests, the correct localization of the ischemic zone. For each test, a multiple choice 6-answer test is given. The criterion of ≥ 90% concordance (at least 18 out of 20 studies) is required, as previously described for first-generation SE multicenter studies [19, 20].
3. Level 3, hub centers read spoke centers studies, consisting in 20 any-quality consecutive studies recorded by the spoke center. The criterion of ≥ 80% concordance (at least 16 out of 20 studies) is required, as previously described for first-generation SE multicenter studies [19].
4. Level 4, core lab reading. All centers should grant full access to images of SE studies entered in the data bank for audit or reading by core lab laboratory, which is the standard for specific subprojects such as number 10 for genetic SE, when every effort needs to be made to minimize variability and a single reader will analyze all studies acquired by different centers, as required by recommendations for small-to-medium sample studies, when resources allow [20].
5. Level 5, specific protocols quality control. Although the SE quality control has proved to work well for regional wall motion analysis, novel SE applications involve different parameters, methodology of acquisition and reading criteria. Therefore, for each subproject, a web-based training session and quality control is organized by the specific hub center and principal investigator to assure consistency of data [21]. The principal investigator of each subproject will prepare a set of 20 studies with rest-stress images. For each test, a multiple choice 6-answers test is given (only 1 correct). The criterion of ≥90% concordance (at least 18 out of 20 studies) is required. The specific signs tested for certification are: end-diastolic and end-systolic volume changes (protocol 1); B-lines (protocol 2, 4, 6 and 9); left ventricular outflow tract gradient (protocol 3 and 10); E/e' (protocol 4); mitral regurgitation quantitative assessment (protocol 5); aortic stenosis quantitative assessment (protocol 5); right ventricular function (protocol 7); pulmonary arterial systolic pressure measurements during stress (protocol 8); coronary flow velocity reserve (protocol 9); left ventricular elastance (protocol 9); global longitudinal strain (protocol 4 and 10).

**Fig. 3:**
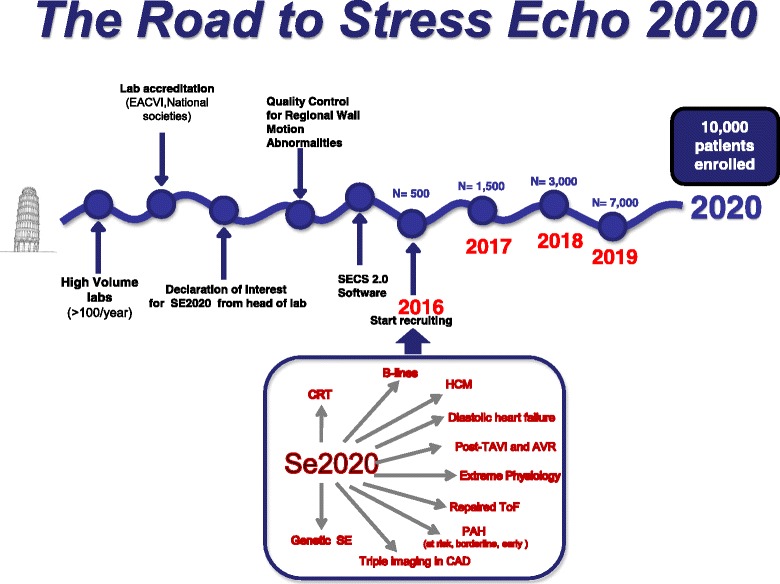
The road to SE2020 for the individual stress echo laboratories. The overall recruitment plan for SE2020 targets 10,000 patients by the end of 2020

This study is also intended as a special level of voluntary accreditation and expertise in the specific field of interest, well above the volume activity criteria requested by guidelines. The accreditation process is run and certified by the Italian scientific society of echocardiography strictly following criteria and procedures of the European association of cardiovascular imaging to ensure standardization and independence of the process. When not otherwise specified, resting and SE measurements are performed according to the latest joint recommendations of European and North-American societies [22]. A simplified view of each lab's road to SE2020 is shown in Fig. 3: the essential pre-requisite is the high-volume activity of the lab, with readers' certification of competence from national or international societies and written declaration of interest in SE2020. After adoption of dedicated SE computerized software by the lab (allowing direct entry of the information in a format compatible with the data bank) and voluntary certification for project-specific SE reading, the center can start recruiting.

### Overall study design

We will collect the experience of Italian, Brazilian, Hungarian and Serbian SE labs over the 5-year period from 2016 to 2020. In this broader framework, 10 sub-projects will address specific patients’ subsets. The target population ranges from 250-patient samples for protocols focused on specific diseases (such as protocol 7 in repaired tetralogy of Fallot) to 2,500 for protocols on heart failure (number 2) to 5,000 to all-comers with known or suspected CAD tested with novel indices (number 9) (Table 1).

**Table 1:** The 10 subprojects

Number	Acronym	Patients	Main parameter	Sample size
1-Cardiac Resynchronization Therapy forecast	CHEF	Prior to cardiac resynchronization therapy	Wall Motion Score Index	500
2-B-lines in heart failure	BHEF	Heart failure	Left ventricular Contractile Rererve	2,500
3-SE in Hypertrophic cardiomyopathy	SEHCA	Hypertrophic cardiomyopathy	Left ventricular outflow tract gradient	250
4- SE in diastolic heart failure	SEDIA	Heart failure preserved ejection fraction	E/e’	250
5-SE in Transvalvular or surgical aortic valve replacement	SETA	After aortic valve replacement	Mitral insufficiency	250
6-SE outdoor	SEO	Extreme exercise	B-lines	250
7-SE in repaired tetralogy of Fallot	SETOF	Repaired Fallot	Tricuspid annular plane systolic excursion	250
8-Doppler SE in pulmonary arterial hypertension	DOPSAH	Pulmonary arterial hypertension (early, borderline, at risk)	Systolic pulmonary arterial pressure	250
9-Diagnosis of CAD by triple imaging SE	DITSE	Known or suspected CAD	Coronary Flow velocity reserve	5,000
10-Genetic SE	GENES	Preclinical dilated or hypertrophic cardiomyopathy	Left ventricular outflow tract gradient	250

Different study projects will cover the entire spectrum of disease, age and clinical status of current patients. The recruited participants are “the wellest of the well” (super-fit athletes entering project 6), the “worried well” (young first-degree relatives of patients with hypertrophic cardiomyopathy or familiar forms of dilated cardiomyopathy or pulmonary arterial hypertension, in project 10), the “suspected sick” (for instance patients with suspected diastolic heart failure or CAD as in projects 4 and 9), up to the "sickest of the sick" (for instance, patients with advanced heart failure or valvular heart disease entering projects 1, 2 and 5). Some degree of overlap is unavoidably present for some projects, for instance with subjects eligible for project 2 who are also recruitable for project 1 (if they undergo cardiac resynchronization therapy) or for project 5 (if they have heart failure with preserved ejection fraction). Over time, patients may move from one project to another: for instance, first-degree relatives of hypertrophic cardiomyopathy patients with negative phenotype enrolled in project 10 may subsequently develop overt forms of disease and be enrolled in project 3. All these potential gray-zone situations will be readily identified in individual SE reports. The investigator is allowed to enter the patient in only one subproject at a given time.

Although the setting will be mainly the Italian cardiological community, all essential documents will be written in English and we plan to extend the project to other communities with long-standing history of cooperation and experience in multicenter trials. Brazilian, Hungarian and Serbian centers are already recruiting and additional laboratories from other countries are now entering the process of accreditation. The project is curiosity-driven, independent from sponsors, and clinically oriented. However, after the planning and start-up phase, support from public or private funding agencies or industries is possible – provided that it is unrestricted and does not interfere in any way with data collection and analysis.

There is no bonus payment for subject recruitment and subject referral. Enrolled patients are referred to the SE lab for clinically-driven indications. Each patient signs an informed consent form allowing scientific utilization of data, respectful of privacy rights, at the time of testing. The study project was submitted by the coordinating center of the principal investigator on January 31, 2016 and approved in its revised form by the Rome-1 ethical committee on July 20, 2016 (protocol number 1487/Lazio1). Ethics committee approval will be sought by each participating center, as needed.

Inclusion criteria shared by all projects are: 1- age < 85 years and > 18 years (except for project 7 regarding repaired Tetralogy of Fallot and project 10 regarding healthy relatives of patients with familial disease, in which children > 10 years can enter the study after parental consent); 2- technically acceptable acoustic window at rest (with at least 14 segments well visualized in at least one projection).

Exclusion criteria shared by all projects are: 1- presence of prognosis-limiting comorbidities, such as advanced cancer, reducing life expectancy to < 1 year; 2- pregnancy/lactation; 3- unwillingness to give informed consent and to enter a regular follow-up program.

SE data will be available to the referring physician.

**Fig. 4:**
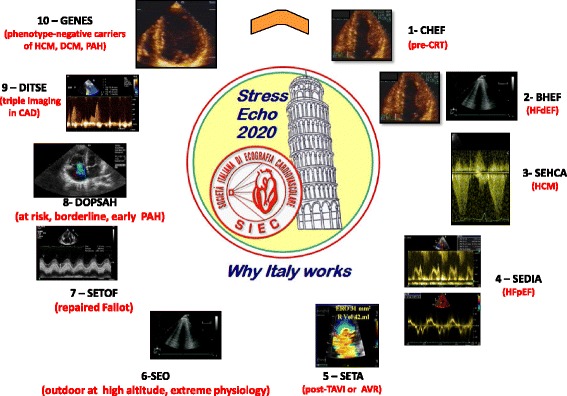
The key echocardiographic parameter for each project, around the logo of SE2020. Clockwise: regional wall motion (project 1, but important in all other projects, mainly project 9); end-systolic volume as part of left ventricular elastance assessment (project 2); left ventricular outflow tract gradient (project 3, but also important in project 10); E/e' ratio (project 4); mitral insufficiency (project 5, but also important in project 2); B-lines (essential in project 6, but also important in project 2, 4 and 9); right ventricular function (project 7); pulmonary hemodynamics (project 8 and 10); regional coronary flow velocity reserve (useful in project 9, but also important in projects 2, 3 and 10)

A brief synopsis of each project is presented for each sub-study, which places emphasis on different parameters tailored on the specific diagnostic question (Fig. 4).

### CHEF

Cardiac resynchronization tHErapy Forecast.

#### Background

Cardiac resynchronization therapy is increasingly used in patients with heart failure, but the identification of “responders” remains challenging, since up to 1 in 3 patients do not show symptomatic improvement with this costly and demanding electrical therapy [10]. QRS width remains the single established criterion to assess intraventricular dyssynchrony according to guidelines; however, there is no accepted consensus on which imaging parameter is best to predict cardiac resynchronization therapy response. Inconsistent results have been obtained with several echocardiographic indices of left ventricular dyssynchrony [23]. The presence of myocardial contractile reserve assessed during SE predicted the response to cardiac resynchronization therapy: the use of SE had obvious potential for a better selection of cardiac resynchronization therapy candidates, in whom a preserved contractility is related to a higher percentage of clinical and echocardiographic responders to cardiac resynchronization therapy [24–27].

#### Aims

The primary aim is to evaluate the feasibility of several indices of SE (including the well- established, such as wall motion score index, and more innovative ones such as left ventricular elastance) in the evaluation of patient candidates for cardiac resynchronization therapy. The secondary aim is to assess the value of each of these parameters (alone or in combination) in predicting the symptomatic and functional improvement in the short-term (1 week to 1 month) after electrical or medical therapy. The tertiary aim is to assess the prognostic value of SE indices for prognostic stratification in the medium-long term.

#### Methods

Patients evaluated prior to cardiac resynchronization therapy, with class I, IIa or IIb for cardiac resynchronization therapy according to ESC 2016 guidelines [10], and therefore with ejection fraction ≤ 35% (by 2-D Simpson method, or real time 3D echocardiography) and QRS duration ≥ 130 ms. Contractile reserve will be assessed during stress (exercise or dobutamine or vasodilators) through variations in Wall Motion Score Index and with more advanced parameters such as left ventricular elastance reserve, as the peak stress/baseline ratio of end-systolic pressure/ end-systolic volume [28]. All other echocardiographic parameters of interest (left ventricular ejection fraction, E/e’, mitral regurgitation, tricuspid annular plane systolic excursion, septal flash, apical rocking, left anterior descending artery coronary flow velocity whenever possible) will also be assessed at baseline and peak stress. All patients will be followed-up within 6-months to 1 year also with resting echocardiographic examination to assess left ventricular remodelling and recovery of function. Any patient excluded from cardiac resynchronization therapy and kept on optimal medical therapy will be included in the follow-up, since the presence of contractile reserve may predict the functional improvement in these patients as well [13].

#### Sample size calculation

If we assume a 60% response rate to cardiac resynchronization therapy, with doubling of likelihood of improvement in presence of a pre-test contractile reserve by SE, with a power of 80% and an alpha error of 5%, a sample size of 277 patients is required. About the same number is required to predict the response to medical therapy in patients eventually not undergoing cardiac resynchronization therapy.

#### Study hypothesis

The presence of contractile reserve is associated with a better prognosis and greater chance of functional recovery with cardiac resynchronization therapy.

### BHEF

Evaluation of B-lines in HEart Failure patients with depressed ejection fraction.

#### Background

B- lines are a semiquantitative sign of extravascular lung water present in 1 out of 3 heart failure patients at rest and in 1 out of 2 during stress, and potentially useful for refining prognostic stratification and titrating diuretic therapy in these patients. Their prognostic power is higher during stress than at rest, and integrates the powerful stratification provided by dynamic assessment of other established predictors such as mitral regurgitation, E/e’ as a surrogate of left ventricular filling pressure, ejection fraction and tricuspid annular plane systolic excursion [29, 30].

#### Aims

The primary aim is to assess the feasibility of several indices of SE (including the well- established such as left ventricular ejection fraction and more innovative ones such as B-lines or left anterior descending coronary flow reserve) in evaluating patients with known or suspected heart failure with reduced ejection fraction. The secondary aim is to assess the value of each of these parameters in predicting the functional impairment, indicated by New York Heart Association class, cardiac natriuretic peptides concentration, peak VO2 and other indices. The tertiary aim is to assess the prognostic value of SE indices for prognostic stratification in the medium-long -term.

#### Methods

We will enrol patients referred to SE (with exercise, dobutamine or dipyridamole) with known or suspected heart failure, with reduced (<40%) ejection fraction. B-lines will be scored with the 28-regions antero-lateral chest assessment as previously described at baseline and immediately after stopping exercise [31]. A simplified 8-region or 4-region scan is also allowed in order to save time without loss of critical information. All other echocardiographic parameters of interest (left ventricular ejection fraction, E/e’, mitral regurgitation, tricuspid annular plane systolic excursion, left anterior descending artery coronary flow velocity whenever possible) will also be assessed at baseline and peak stress [32]. All patients will be followed-up for at least 1 year.

#### Sample size calculation

If we conservatively assume a 30% yearly incidence of composite end-points (death, myocardial infarction, new hospital readmission, heart transplant, ventricular assist device implantation, aborted sudden death) and a 5% incidence of death, with doubling of likelihood of events in presence of a positive SE (for B-lines increase during stress or lack of contractile reserve or severe mitral insufficiency), with a power of 95% and an alpha error of 5%, with an attrition rate of 15%, a sample size of about 2500 patients is required if the effect on mortality is evaluated.

#### Study hypothesis

The integrated assessment of 5 major echocardiographic variables during stress adds power to the prognostic stratification operated by isolated echocardiographic predictors in heart failure patients. The prognostically meaningful signs explore 5 key links in the patho-physiologic chain behind cardiovascular response in heart failure: left ventricular systolic function – left ventricular diastolic function - mitral valve regurgitation - lung water accumulation - right ventricular function.

### SEHCA

Stress Echo in Hypertrophic Cardiomyopathy.

#### Background

The impact of SE in hypertrophic cardiomyopathy is limited by lack of standardization and outcome data. Current guidelines recommend SE solely for evaluation of left ventricular outflow tract obstruction [33]. However, large-scale registry data show that SE positivity for ischemic criteria (such as new wall motion abnormalities and coronary flow velocity reserve) rather than inducible gradients predict adverse outcome in hypertrophic cardiomyopathy [34]. Thus, important prognostic as well as functional information may be derived from SE, although their clinical impact remains limited due lack of standardized data collection and uniform protocols. Currently there are virtually no two centers performing SE in the same way in hypertrophic cardiomyopathy patients, and the test remains grossly underutilized due to (unjustified) concerns with safety. Prospective, standardized, multicenter data collection is necessary to achieve comprehensive evaluation of SE potential in this disease. Of note, exercise limitation and breathlessness may be due to a number of different causes, including left ventricular outflow tract obstruction (which may increase, remain stable or decrease during exercise), functional mitral regurgitation, restrictive physiology, amplified mechanical dyssynchrony, coronary microvascular disease or – less frequently – ischemia associated with prognostically unfavorable stress-induced wall motion abnormalities. Despite similar clinical manifestations, management may differ substantially based on the mechanisms [35]. SE is the only test with the potential to discriminate the various components, allowing a targeted treatment driven by pathophysiology, but prospective data are required to substantiate this hypothesis. Of note, general consensus exists regarding the advisability of preferring physiological provocation with exercise over pharmacological stressors (particularly dobutamine due to substantial prevalence of false positives with regard to inducible obstruction).

#### Aims

The primary aim is to evaluate the feasibility of several indices of SE (from the well- established such as left ventricular outflow tract gradient to more innovative features such as B-lines or left anterior descending coronary flow reserve) in the evaluation of hypertrophic cardiomyopathy patients. The secondary aim is to assess the value of each of these parameters in predicting functional impairment, indicated by New York Heart Association class, European Society of Cardiology sudden cardiac death risk score and other indices. The tertiary aim is to assess the prognostic value of SE indices for prognostic stratification in the medium-long-term.

#### Methods

Diagnosis of hypertrophic cardiomyopathy will be based on existing guidelines [33]. Phenocopies such as infiltrative/storage disease (eg, Fabry) will be excluded. Low-to-intermediate risk symptomatic or asymptomatic hypertrophic cardiomyopathy patients will undergo exercise SE with assessment at each stage and during recovery of wall motion, mitral insufficiency, left ventricular outflow tract gradient (in orthostatic position) and E/e’. If feasible, coronary flow velocity reserve on left anterior descending and lung ultrasound B-lines will also be assessed. Only when patients are unable to exercise, or for the specific aim of assessing coronary flow velocity reserve, will a vasodilator stress (high dose dipyridamole or adenosine without atropine) be performed. All patients will be followed-up and the prognostic value of different rest and SE parameters (also compared to standard prognostic indices) will be assessed.

#### Sample size calculation

The composite end-point of the study comprises death (cardiovascular and all-cause), new hospital admission for acute heart failure, newly onset atrial fibrillation, aborted sudden death, heart transplant or ventricular assist device implantation. A pilot study showed an incidence of events around 8% per year [34]. If we assume a positivity rate to SE (by composite criteria) of 40%, with doubling of likelihood of events in presence of SE positivity (by any criteria), with a power of 80%, an alpha error of 5%, and an attrition rate of 10%, a sample size of about 250 patients is required.

#### Study hypothesis

Hypertrophic cardiomyopathy patients with inducible wall motion abnormalities and reduced coronary flow velocity reserve (or lung ultrasound B-lines) are at substantially higher risk for subsequent unfavorable events than patients with normal wall motion and preserved coronary flow velocity reserve. Exercise capacity predicts outcome in hypertrophic cardiomyopathy patients independent of the presence and magnitude of resting or provocable obstruction.

### SEDIA

Stress Echo in Diastolic Heart failure.

#### Background

Diastolic SE should be considered in the breathless patient with normal left ventricular ejection fraction, high cardiac natriuretic peptides and low exercise tolerance, especially in presence of cardiovascular risk factors (advanced age, arterial systemic hypertension, diabetes mellitus, obesity and sedentary lifestyle) after ruling out other common cardiac (heart valve disease, coronary artery disease) and non-cardiac causes of dyspnea (mainly anemia and chronic obstructive pulmonary disease). « Red flag » indicators of possible diastolic dysfunction include left ventricular hypertrophy, enlarged left atrium and pulmonary hypertension. During SE, findings which may account for unexplained dyspnea beyond diastolic dysfunction are left ventricular outflow tract obstruction, dynamic mitral regurgitation, and new onset regional wall motion abnormalities [36]. The diagnostic SE criteria of heart failure with preserved ejection fraction are reduced stroke volume and cardiac output reserve, high left ventricular filling pressures (average E/e’ ratio > 14) and pulmonary hypertension, which also identify patients with more severe prognosis [37, 38]. In patients with heart failure and either preserved (>50%) or mid-range (40-49%) ejection fraction, SE may allow the identification of diastolic dysfunction in patients with parameters inconclusive at rest [10]. According to the latest 2016 recommendations on the evaluation of left ventricular diastolic function by echocardiography [36], diastolic stress testing would not be indicated in patients with either completely normal diastolic function at rest (in whom a severe diastolic dysfunction is unlikely to develop during stress) and in patients with ≥ grade 2 diastolic dysfunction at rest (with elevated filling pressures at rest which will likely increase further during exercise). However, testing of these patients can be valuable to assess the incremental value, if any, of newer candidate indices such as left ventricular end-diastolic volume reserve and B-lines. In addition, also patients with normal left ventricular diastolic function at rest can indeed develop increase in left ventricular filling pressures with dyspnea when exercising.

#### Aims

The primary aim is to evaluate the feasibility of several indices of SE (including more established such as E/e' ratio and more innovative ones such as B-lines or left ventricular diastolic volume reserve) in the evaluation of patients with known or suspected heart failure with preserved or mid-range ejection fraction. The secondary aim is to assess the value of each of these parameters in predicting the functional impairment, indicated by New York Heart Association class, cardiac natriuretic peptides concentration, peak VO2 and other indices. The tertiary aim is to assess the prognostic value of SE indices for prognostic stratification of heart failure with preserved ejection fraction in the medium-long-term.

#### Methods

Patients with known or suspected heart failure with preserved ejection fraction by 2016 ESC criteria will be enrolled and studied with cycle-ergometer in semi-supine SE (or treadmill). The test is especially indicated in patients with dyspnea and grade 1 diastolic dysfunction at rest, identified as mitral E/A ratio ≤ 0.8, average E/e' ratio < 10, peak tricuspid regurgitant jet velocity <2.8 m/s and left atrium volume index normal (<34 mL/m^2^) or increased [36]. In patients unable to exercise, pharmacological test of choice (dobutamine or vasodilator) is allowed, but not recommended. The diastolic assessment should be included into all exercise SE tests by measuring standard Doppler-derived mitral inflow velocity, pulsed Tissue Doppler of mitral annulus, and retrograde tricuspid gradient of tricuspid regurgitation. These measurements can be performed at intermediate load of exercise and/or 1- 2 min after the end of the exercise, after obtaining wall motion acquisitions, when the heart rate decreases and mitral inflow E and A velocities appear to be well separated. As a part of the “diastolic package”, we will also assess, at baseline, intermediate load (50 watts) and peak-post stress [36]: diastolic left ventricular volume index (to evaluate left ventricular diastolic volume reserve, impaired in stiff hearts, which are less dilated for any given filling pressure); systolic left ventricular volume index (for assessment of left ventricular elastance, which may unmask occult systolic dysfunction with normal ejection fraction increase); ejection fraction and both stroke volume and cardiac output (to assess conventional contractile reserve); mitral regurgitation and left ventricular outflow tract obstruction; pulmonary artery systolic pressure (from velocity of tricuspid regurgitation); B-lines during stress (to provide a direct imaging of extra-vascular lung water accumulation as a direct cause of dyspnea), since they can be present at rest in diastolic heart failure [32]. Global longitudinal strain could be optionally determined as the average of the regional longitudinal strain measured in 17-segments model from the apical long-axis, 4-chamber and 2-chamber view. Despite not being independent on preload and afterload, global longitudinal strain could be considered as an appropriate, alternative parameter of left ventricular contractile reserve [22, 39]. On the basis of currently accepted criteria, the test is considered positive for diastolic dysfunction when all of the following three conditions are met during exercise: average E/e' > 14 or septal E/e' ratio > 15, peak tricuspid regurgitant jet velocity >2.8 m/s and septal e' velocity < 7 cm/s at baseline [36].

#### Sample size calculation

The composite end-point of the study comprises death (cardiovascular and all-cause), new hospital admission for acute heart failure, aborted sudden death, heart transplant or ventricular assist device implantation. The expected incidence of events is around 20% per year [10]. We assume a positivity rate to SE (by composite criteria: increase in E/e' and/or increase in pulmonary artery systolic pressure and/ or decrease in septal e' velocity and/or increase in B-lines) of 30%, with doubling of likelihood of events in presence of SE positivity (by any criteria). With a power of 80%, an alpha error of 5%, and an attrition rate of 10% (for exams non-feasible and/ or for exclusion criteria during the screening phase due to previously unrecognized dynamic severe mitral regurgitation or left ventricular outflow obstruction), a sample size of about 250 patients is required.

#### Study hypothesis

In patients with known or suspected heart failure with reduced ejection fraction, higher left ventricular filling pressures, more B- lines and lower increases in end-diastolic volumes during stress are associated with worse prognosis.

### SETA

Stress Echo in Transcatheter or surgical Aortic Valve Implantation.

#### Background

Transcatheter Aortic Valve Implantation is an extraordinarily effective but still a relatively novel technology, and short and long term morbidity and mortality after Transcatheter Aortic Valve Implantation remains significant. There is substantial interest in the identification and modification of factors influencing prognosis before and after the procedure. The severity of concomitant mitral regurgitation improves after the procedure in 2 out of 3 patients, but baseline moderate-severe mitral regurgitation and significant residual mitral regurgitation are associated with an increase in mortality after Transcatheter Aortic Valve Implantation and represent an important group to target with medical or transcatheter therapies in the future [40]. Stress echo plays a pivotal role in valvular heart disease [41], but its role after either Transcatheter Aortic Valve Implantation or surgical replacement is still unsettled, although it has potential to identify the presence and severity of mitral regurgitation and aortic stenosis after intervention, so as to refine the prognostic strategy and better tailor treatment.

#### Aims

The primary aim is to evaluate the feasibility of SE focused on mitral and aortic reserve after aortic valve replacement (with Transcatheter or surgical aortic valve implantation). The secondary aim is to assess the presence and entity of changes in valvular and ventricular function and their correlation with indices of functional severity (New York Heart Association Class, cardiac natriuretic peptides, peak VO_2_, etc.).. The tertiary aim is to assess the prognostic value of SE indices for prognostic stratification in the medium-long-term.

#### Methods

Patients with previous (from 6 months to 10 years) surgical or Transcatheter Aortic Valve Implantation capable of exercising and with absent-to-moderate mitral insufficiency will be enrolled and studied with semisupine SE. The full quantitative evaluation of mitral regurgitation and aortic stenosis will be performed according to recommendations of the European Society of Cardiology. In addition, assessment of B- lines (as in protocol 2), right ventricular function (as in protocol 7), left ventricular elastance (as in protocol 1) will be performed at baseline and peak stress. Exercise will be started at 15 watts, with 5-min steps and 15-watt increments per step [13]. In patients unable to exercise, a pharmacological test of choice (dobutamine or vasodilator) is allowed, but not recommended.

#### Sample size calculation

The expected incidence of SE positivity (by composite criteria: increase in mean transaortic gradient > 20 mmHg and/or increase in mitral insufficiency > 1 grade and/or increase in B-lines and/or increase in systolic pulmonary artery pressure) is around 30% post- Transcatheter Aortic Valve Implantation or aortic valve surgical replacement [10]. With a power of 80%, an alpha error of 5%, and an attrition rate of 10%, a sample size of about 100 patients is required to detect a significant stress-induced increase in mitral regurgitation severity. For the prognostic analysis (tertiary end-point). If we conservatively assume a 20% yearly incidence of composite end-points (death, myocardial infarction, new hospital readmission, heart transplant, ventricular assist device implantation, aborted sudden death), with doubling of likelihood of events in presence of a positive SE (for severe mitral insufficiency and/or abnormally increased transaortic gradient and/ or B-lines and/ or abnormal pulmonary artery systolic pressure), with a power of 80% and an alpha error of 5%, a sample size of about 250 patients is required with a 3 -year follow-up.

#### Study hypothesis

In patients with absent-to moderate mitral regurgitation in resting transthoracic echocardiography after either surgical or Transcatheter Aortic Valve Implantation, those with lower transaortic gradient and less mitral regurgitation during stress will have a more favourable outcome than patients with higher gradients and more severe residual mitral regurgitation during stress.

### SEO

Stress Echo Outdoor in Extreme conditions.

#### Background

SE with B-lines can also be performed outdoors, with pocket size or portable instruments, in a setting of ecological stress entirely different from standard indoor testing [4]. The diagnostic target is the diagnosis, or early subclinical identification, of life-threatening disease at high altitudes or any kind of environmental pulmonary edema. In this challenging but fascinating context, lung ultrasound detects B-lines in 20 to 40% of normal and/or super-fit subjects in extreme physiology settings, such as high altitude [42, 43], deep underwater apnea diving [44] or endurance exercise [45].

#### Aims

The primary aim is to evaluate the feasibility of outdoor SE focused on B-lines in the logistic setting of extreme physiology. The secondary aim is to assess the presence and amount of B-lines increase at peak stress vs baseline conditions (pre-exercise; pre-apnea; pre-ascent) and to correlate lung ultrasound findings with symptoms (such as dyspnea, cough, fatigue). The tertiary aim is to assess the prognostic value of SE indices for predicting spontaneously occurring pulmonary edema.

#### Methods

Subjects involved in extreme sporting events (competitive triathlon, marathon, apnea diving etc.) or ordinary exercise in extreme environments (trekking at high altitude) will undergo lung ultrasound scan for B-lines before, soon after (within 10 min) and (when positive soon after) later after (6 to 24 h) the acute extreme exercise. Additional clinical information will be collected in addition to standard case report form (including Acute Mountain Sickness scores in subjects evaluated at high altitude), and will include details on type and duration of exercise (apnea diving vs ascent trekking vs strenuous exercise at sea level etc.) [43]; environmental conditions (temperature, humidity, setting, wind etc.); location and timing of scanning, with possibility of limited scanning (8 regions instead of the standard 28) in more hostile environments.

#### Sample size calculation

The expected incidence of SE positivity (increase in B-lines >5 compared to rest) is around 30% [43]. With a power of 80%, an alpha error of 5%, and an attrition rate of 10%, a sample size of about 80 patients is required to detect a significant stress-induced increase in B-lines in each of the three major study subgroups: high altitude trekkers (*n* = 100); marathon and ultra marathon runners (*n* = 80) and apnea divers (*n* = 70).

#### Study hypothesis

Asymptomatic subjects with evidence of B- lines are more likely to develop clinically overt forms of environmental pulmonary edema with persistence of exposure or in the future when re-challenged under similar conditions.

### SETOF

Stress Echo in operated Tetralogy of Fallot.

#### Background

Tetralogy of Fallot is the most common cyanotic congenital heart lesion, and since treatments became available over 70 years ago, there are now a large number of patients with repaired Tetralogy of Fallot [46]. After Tetralogy of Fallot repair, children often have residual lesions (the most common being pulmonary regurgitation) which can be treated with surgical or catheter-based pulmonary valve replacement decreasing right ventricular size but not yet correlated with improved outcome [46]. Pulmonary regurgitation can cause progressive right ventricular dilatation and dysfunction. In Tetralogy of Fallot patients morbidity and mortality are strongly related to right ventricular dysfunction. For this reason, the early detection of right ventricular dysfunction before it reaches an irreversible stage remains crucial [47]. Unfortunately, resting parameters have shown a limited ability to detect early impairment of right ventricular function. Recently, a few studies have suggested that physical or pharmacological stress may unmask abnormalities of right ventricular function in patients with repaired Tetralogy of Fallot, with normal right ventricular function under resting conditions [48–50]. Physical exercise SE allows the simultaneous assessment of right and left ventricular global and regional function and Doppler parameters [51].

#### Aims

The primary aim is to evaluate the feasibility of right ventricular SE in patients with repaired tetralogy of Fallot. The secondary aim is to assess the presence and amount of right ventricular contractile reserve and its correlation with indices of functional severity (NYHA class, cardiac natriuretic peptides, peak VO_2_, 6-min walking test, etc.). The tertiary aim is to assess the prognostic value of SE indices for prognostic stratification in the medium and long-term.

#### Methods

Patients with repaired Tetralogy of Fallot or Fallot-like pathology (double-outlet right ventricle Fallot type, tetralogy of Fallot with pulmonary atresia), evaluated at least 1 year after the last surgical or percutaneous procedure, will be recruited by regional reference centers for congenital heart disease. Additional inclusion criteria are age > 10 years, height > 140 cm, New York Heart Association class I or II. Right ventricular function will be assessed at baseline and peak stress with variations (rest and peak stress) of tricuspid annular plane systolic excursion, an index of right ventricular longitudinal function, and right ventricular fractional area change (a load-dependent index of right ventricular inlet function). Due to the influence of load on these measures, they tend to reflect right ventricular arterial coupling rather than measures of right ventricular contractility per se. To distinguish between genuine right ventricular dysfunction and/or pathological increases in pulmonary vascular load, whenever possible we will combine systolic pulmonary artery pressure and right ventricular end-systolic area using echocardiography to calculate right ventricular end-systolic pressure-area relation as a surrogate of right ventricular contractility [52].

Peak systolic tricuspid annulus velocity and conventional indices of left ventricular systolic and diastolic function will also be measured at baseline and peak stress. Left ventricular function will also be assessed through measurement of ejection fraction, wall motion score index and E/e' at baseline and peak stress.

#### Sample size calculation

The expected incidence of SE positivity (by increase in tricuspid annular plane systolic excursion < 5 mm) is around 30% as shown by previous pilot studies [10]. With a power of 80%, an alpha error of 5%, and an attrition rate of 10%, a sample size of about 250 patients is required to detect a significant stress-induced increase in tricuspid annular plane systolic excursion. For the exploratory prognostic analysis (tertiary end-point), we conservatively assume a 20% yearly incidence of the pre-determined end-point (death, myocardial infarction, new hospital readmission, heart transplant, ventricular assist device implantation, aborted sudden death), with doubling of likelihood of events in presence of a positive SE (reduced right ventricular contractile reserve). With a power of 80% and an alpha error of 5%, a sample size of about 250 patients is required with a 3 -year follow-up.

#### Study hypothesis

Repaired tetralogy of Fallot patients with better right (and possibly left) ventricular reserve will have less chance of developing adverse events in their natural history.

### DOSPAH

Doppler Stress echo in Pulmonary Arterial Hypertension.

#### Background

Patients at risk of pulmonary arterial hypertension at rest may show abnormal flow-adjusted increase in pulmonary pressures during exercise and are more likely to develop subsequent resting pulmonary hypertension [53, 54]. In patients with established or borderline pulmonary arterial hypertension capable of exercising, the level of exercise-induced increase in systolic pulmonary artery pressure and a reduced right ventricular contractile reserve are associated with a poorer prognosis [55]. The potential value of exercise-stress echo can be diagnostic in patients at risk of pulmonary arterial hypertension, and prognostic in patients with early established or borderline pulmonary arterial hypertension (Group 1 of European Society of Cardiology guidelines 2015) [56].

#### Aims

The primary aim is to evaluate the feasibility of SE focused on pulmonary hemodynamics and right ventricular function in patients at risk, borderline or early established pulmonary arterial hypertension. The secondary aim is to assess the presence and amount of right ventricular contractile reserve and its correlation with indices of functional severity (New York Heart Association Class, cardiac natriuretic peptides, peak VO_2_, etc.). The tertiary aim is to assess the prognostic value of SE for predicting increase of resting systolic pulmonary artery pressure in the medium-term (2-years follow-up).

#### Methods

Patients at risk, borderline, or early established pulmonary hypertension capable of exercising will be recruited by regional reference centers for pulmonary hypertension. A physical stress with exercise will be performed. A thorough non-invasive hemodynamic assessment will be performed, including evaluation of rest and peak stress of: 1) Systolic pulmonary artery pressure from maximal velocity of tricuspid Doppler regurgitant jet adding the value of the right atrial pressure estimated on the basis of diameter and inspiratory collapse index of the inferior vena cava [57]; 2) mean pulmonary artery pressure as 0.6 x systolic pulmonary artery pressure +2 [58]; 3) cardiac output from the time-velocity integral of the left ventricular outflow tract [59]. SE positivity criteria will be the absolute increase in systolic pulmonary artery pressure >50 mmHg or the delta mean pulmonary arterial pressure/cardiac output > 3 mmHg/L/min. When a good-quality tricuspid jet signal cannot be sampled, the mean pulmonary artery pressure will be estimated based on pulsed-Doppler measurement of the acceleration time of pulmonary flow, sampled at the right ventricular outflow tract as mean pulmonary arterial pressure =79 - (0.6 x Acceleration time) [60]. Right ventricular contractile reserve will be measured as described above in project 7.

#### Sample size calculation

The expected incidence of SE positivity is around 30% as shown by previous pilot studies [10]. With a power of 80%, an alpha error of 5%, and an attrition rate of 10%, a sample size of about 250 patients is required to detect a significant stress-induced hemodynamic changes. For the prognostic analysis (tertiary end-point), if we conservatively assume a 15% yearly incidence of the pre-determined end-point (increase in resting systolic pulmonary artery pressure > 35 mmHg), with doubling of likelihood of events in presence of a positive SE, with a power of 80% and an alpha error of 5%, a sample size of about 250 patients is required with a 3 -year follow-up.

#### Study hypothesis

In subjects at risk of pulmonary arterial hypertension, those with higher pulmonary artery systolic pressure and lower decrease in pulmonary vascular resistance (as delta mean pulmonary arterial pressure/cardiac output >3 mmHg/L/min) during exercise will have more chance of developing resting pulmonary hypertension in their natural history [59, 60]. In patients with established early or borderline pulmonary arterial hypertension, those with lower decrease in pulmonary vascular resistances and worse right ventricular contractile reserve during exercise will have more chances of developing adverse events in their natural history.

### DITSE

Diagnosis of CAD by Triple imaging Stress Echo (wall motion, coronary flow reserve and left ventricular elastance) plus B-lines.

#### Background

The cornerstone of diagnosis with SE is the finding of reversible regional wall motion abnormalities. However, the potentially valuable, diagnostic and prognostic, information provided by SE extends well beyond regional wall motion. In vasodilator and also during exercise stress, a clear step-up in diagnostic sensitivity (with a modest loss in specificity) and risk stratification capability is obtained with assessment of coronary flow velocity reserve in the left anterior descending coronary artery [61–63]. In exercise and dobutamine, and also with vasodilator stress, critical gains in sensitivity can be achieved by non invasive assessment of left ventricular contractile reserve through changes in left ventricular elastance, a load-independent index of left ventricular contractility more diagnostically and prognostically valuable than changes in ejection fraction [64, 65]. Normal values of coronary flow velocity reserve are >2.0 for all stresses, while the contractile reserve normal values are >2.0 for exercise and dobutamine but >1.0 for vasodilator stress. Furthermore, evaluation of B-lines can introduce a variable of additional prognostic value indicating acute accumulation of extra-vascular lung water [30, 31].

#### Aims

The *primary* aim is to evaluate the feasibility (with different stresses and protocol, with or without contrast) of rest and stress-induced integrated approach during SE with the “quadruple imaging” approach: regional wall motion abnormalities (standard, *single imaging* approach), coronary flow velocity reserve on left anterior descending (advanced, *dual imaging* approach); left ventricular elastance (derived from simple raw measures of cuff sphygmomanometer systolic arterial pressures/end-systolic volume, *triple imaging* approach); extra-vascular lung water (derived from B-lines from lung sonography, *quadruple* imaging approach). The *secondary* aim is to assess the diagnostic value of each of these parameters (alone and in combination) in predicting underlying coronary anatomy independently assessed by cardiac computed tomography and/or invasive coronary angiography (required when cardiac computed tomography is positive). The *tertiary* aim is to assess the prognostic value of the integrated SE in predicting events in the medium-term follow-up (up to 2 years)

#### Methods

All patients (“*allcomers*”) referred to the SE lab with suspected CAD (history of chest pain or asymptomatic with previous positivity of any stress test, different from SE) will be evaluated with standard regional wall motion analysis and also – whenever feasible - with left ventricular coronary flow reserve (at least on the left anterior descending coronary artery) and left ventricular elastance reserve (whatever the stress: exercise, vasodilator or dobutamine). B-lines can be assessed at baseline and soon after stress. For each stress, all the four indices (regional wall motion, coronary flow velocity reserve, left ventricular elastance reserve and B-lines) can be obtained, if possible. For coronary flow velocity reserve assessment on left anterior descending, both the maximum diastolic flow velocity and, when possible, the whole envelope to extrapolate the new parameter labelled coronary functional reserve will be measured. Also patients with known CAD referred to SE for prognostic stratification will be included and evaluated for prognostic outcome, including (when feasible) resting transthoracic echocardiography evaluation of regional and global left ventricular function to assess progression to left ventricular dilation and dysfunction (ejection fraction decrease of > 15% and below 35%).

Centers can recruit with any combination of dual imaging (regional wall motion plus at least one of the three more innovative parameters: coronary flow velocity reserve, left ventricular contractile reserve and B-lines) as dictated by the locally available technology and expertise. According to preliminary experience, the feasibility rate is expected to be highest for left ventricular contractile reserve and B-lines, and lower for more demanding coronary flow velocity reserve.

#### Sample size calculation

The expected incidence of SE positivity (by composite criteria: regional wall motion abnormalities in 10%; reduction in coronary flow reserve velocity in 30%, blunted left ventricular contractile reserve in 20%; B-line increase >5) is around 35% [10]. A subset of 500 patients with coronary angiography verification (by invasive angiography or coronary CT) is required for reliable estimates of feasibility, imaging time and analysis time of each parameter. For prognostic tertiary end-point, if we conservatively assume a 5% yearly incidence of composite end-points (death, myocardial infarction, new hospital readmission, heart transplant, ventricular assist device implantation, aborted sudden death), with doubling of likelihood of events in presence of a positive SE (for composite criteria), with a power of 90% and an alpha error of 5%, a sample size of about 2500 patients is required with a 3-year follow-up. If only mortality is considered, a sample size of about 5,000 patients will be required.

#### Study hypothesis

Quadruple imaging combining coronary flow velocity reserve on left anterior descending artery, left ventricular contractility reserve evaluation with elastance assessment and B-lines in addition to conventional regional wall motion analysis is feasible with reasonable success rate, and diagnostically useful in all patients with all stresses. Even in the absence of inducible regional wall motion abnormalities, a lower coronary flow velocity reserve on left anterior descending coronary artery and/or a reduced left ventricular contractile reserve and/or an increase in B-lines will identify patients with worse outcome. When feasible, the combination of the quadruple imaging (regional wall motion, coronary flow reserve, left ventricular elastance reserve and B-lines) yields more prognostic information than any of the four parameters considered alone.

### GENES

Genetic Stress echocardiography.

#### Background

The identification of phenotype-negative and genotype positive carriers of pathologic mutations is an important, yet still elusive, target for clinical cardiologists, although encouraging preliminary results have been reported with SE allowing identification of dynamic gradients in hypertrophic cardiomyopathy mutation carriers prior to development of hypertrophy [66], increased pulmonary resistance in mutation carriers of familial pulmonary hypertension with normal pulmonary pressure at rest [67], and higher resting end-diastolic volumes and possibly reduced contractile reserve during stress in familial dilated cardiomyopathy mutation carriers with normal left ventricular function at rest [68].

#### Aims

The primary aim is to evaluate the feasibility of SE in genetically characterized (on the basis of existing recommendations) first-degree relatives of patients with genetically transmitted cardiac diseases (such as hypertrophic cardiomyopathy, familial pulmonary arterial hypertension, and familial dilated cardiomyopathy). The secondary aim is to assess the value of disease-specific sentinel-parameters during SE in predicting the carrier and non-carrier status of healthy asymptomatic relatives, with the carrier status predicted by increased left ventricular outflow tract gradient in hypertrophic cardiomyopathy, exaggerated rise in systolic pulmonary arterial pressure in familial pulmonary arterial hypertension, and by blunted left ventricular contractile reserve in familial dilated cardiomyopathy. The tertiary, exploratory aim is to assess the value of SE indices for predicting the phenotypic expression of the disease in the medium-long term.

#### Methods

We will initially select 75 patients (25 for each disease) with documented disease and mutant gene identified by next-generation sequencing platform using targeted disease gene panels in tertiary care centers of genetic cardiology (in Florence-Careggi, Naples-Monaldi, Pisa-CNR or Cattinara-Trieste); they will undergo standardized SE testing. Furthermore, we will enroll around 250 first-degree relatives of the initially considered probands, with similar number for each disease, all with good-quality echocardiographic imaging, normal findings at rest and age range preferentially between 10 and 21 years (since a pathological phenotype is more likely to develop in the following 5 years when a diseased genotype is present). The relatives will undergo both genetic testing for the gene identified in the proband and SE testing with centralized core lab reading by observers blinded to genetic testing results. Each SE testing will be tailored on the specific question: hypertrophic cardiomyopathy as in protocol 3 (primary endpoint: orthostatic exercise induced change in left ventricular outflow tract gradient); pulmonary hypertension as in protocol 8 (primary endpoint: rest-exercise, flow-adjusted, variation in pulmonary vascular resistances); dilated cardiomyopathy as in protocol 1 (primary endpoint: left ventricular elastance change following exercise). All enrolled subjects (both genotype-positive and genotype-negative) will undergo yearly clinical and resting transthoracic echocardiography follow-up. The criteria for disease detection in these individuals include minimal phenotypes with low cut-off values (a wall thickness > 13 mm in the anterior septum and/or posterior left ventricular wall for hypertrophic cardiomyopathy; resting pulmonary artery systolic pressure > 40 mmHg verified by cath lab for primary pulmonary hypertension; a dilated left ventricular end-diastolic volume index  > 74 mL/m^2^ for men or 61 mL/m^2^ for women and/or ejection fraction < 45% for dilated cardiomyopathy) [22]. In all subjects, coronary flow reserve during exercise will be evaluated, since a coronary microvascular abnormality has been reported as a very early finding in both dilated and hypertrophic cardiomyopathy, and this technique, albeit demanding, is feasible with last generation technology also during exercise [63].

#### Sample size calculation

We conservatively assume a significant increase in left ventricular outflow tract gradient > 50 mmHg during exercise in 50% of mutation carriers (as shown in a pilot study, 66) versus 5% of non-carriers. A significant difference in prevalence of left ventricular outflow tract gradient will be observed, with a power of 80% and an alpha error of 5% with an attrition rate of 10%, with a sample size of about 80 patients for the hypertrophic cardiomyopathy study. A similar sample size estimation is applied to the other two subprojects of familial dilated cardiomyopathy (primary SE endpoint: increase in left ventricular contractile reserve <2.0) and familial pulmonary hypertension (primary SE endpoint: increase in systolic pulmonary artery pressure > 50 mmHg), with a total sample size of about 250 subjects for the whole subproject.

#### Study hypothesis

SE abnormalities predict carrier status in families with genetic cardiomyopathies. In genetic carriers, SE abnormalities may predict development of overt disease at mid-term follow-up.

## Discussion

The overarching aim of SE2020 is to provide data directly relevant to patient care by filling the existing evidence gap in several areas of SE. Due to health care rationing and shortage of resources for independent, patient-oriented research, the SE community should face the challenge to optimize the use of infrastructural resources to provide the evidence base necessary for tailoring the right SE to the right patient with the right technology, used by the right (properly trained and certified) cardiologist.

There are several possible kinds of added values in this project.

### Clinical

Only multicenter trials can provide the necessary information for validation of any diagnostic procedure in a reasonable amount of time; otherwise, tests that are hazardous or unfeasible, or both, may become accepted before inadequacies are recognized. SE is widely validated and has stood the test of time, but this is true mainly (if not only) for CAD and heart failure applications based on regional wall motion abnormalities. New applications are conceptually innovative, based on various parameters, applied to different patients, and we cannot skip the chain of validation required to transform a promising innovation into an established procedure [3, 4].

### Scientific

Enrollment of patients in 10 different studies of special interest will provide a considerable amount of unique information in a relatively short time in several areas of critical scientific interest. In particular, in all of these fields available prognostic data are absent or, when existent, suffer from relatively small sample size and include soft and heterogeneous end-points to document prognostic power. The study will also generate an intellectual and professional network which is the ideal platform for future randomized, outcome intervention trials to assess the value of SE-based interventions, which is the highest level of evidence required by guidelines to change current clinical practice and yet lacking to date for several new applications of cardiac imaging [3, 4].

### Educational

With a coordinated effort endorsed by the Italian Society of Cardiovascular Echography, the stress echo community will develop, test in the field and disseminate a structured software for collecting medical history, demographic information, clinical, SE, other imaging techniques, and outcome data for patients entering the standardized Italian and international “SE lab without walls”. The participants to this SE lab will voluntarily agree to share a common language of indications, execution, reporting, image storage and archiving SE information performed in accredited centers, which previously underwent quality control of reading for specific SE skills. This will help to advance the field of SE, minimizing the greatest weakness of the technique, i.e., inter-laboratory variability in reading criteria, reporting heterogeneity and lack of permanent quality control standards [16, 17].

### Economic

Today SE enjoys the advantages of economic sustainability and lack of radiation, making it potentially dominant in the current era of health care rationing [69]. However, to be cost-effective the indication must be appropriate, the exam must be performed by trained personnel, and with robust evidence supporting its specific clinical use. Otherwise, SE becomes just another tree in the cardiac imaging forest of inappropriate and redundant testing [70].

### Comparison with previous studies

The same conceptual and operative template of SE2020 was put in place almost 30 years ago, at the beginning of the SE era, when the Italian first-generation multicenter studies provided unique evidences for the use of pharmacological testing with dipyridamole (EPIC; Echo-Persantine International Cooperative) study and dobutamine (EDIC, Echo-Dobutamine International Cooperative) study for the diagnosis of coronary artery disease. They addressed key aspects of stress testing such as safety and prognostic value in specific patient subsets [71–74]. From 1992 to 2012, over 30 articles from this multicenter trial network were published in top peer-reviewed journals and more importantly, these results rapidly shaped clinical practice and scientific guidelines [5, 6], since practice is easier to change when based on evidence that someone contributed to building in his/her own laboratory. In the footsteps of this template, the same paradigm will be followed today with SE2020 (Table 2). At that time, the main focus was on CAD; today, on conditions beyond CAD. Yesterday, the key sign was wall motion with 2-D, today an array of disease-specific diagnostic markers (from B-lines with lung ultrasound to coronary flow reserve with pulsed Doppler). At that time, early adopters were interested mainly for scientific reasons; today, virtually every lab can play a role, and is motivated by clinical interest to share experiences and standardize languages in a widely deregulated field. Yesterday, the driving force and core team of investigators came from a research institute (Italian National Research Council) with a top-down approach, from a research vision; today, from a scientific society (Italian Society of Echocardiography) networking all interested clinical cardiologists with an inclusive approach generating a bottom-up strategy, where all different expertise is added in a common intellectual architecture. The rationale of first- and second-generation studies is the same. As scientists and as clinicians, we need to act on the basis of effectiveness, real-world data populated by real patients, real doctors and real problems rather than on published efficacy data collected in ideal conditions but not always representative of true life. The seed of efficacy should not be mistaken for the fruit of effectiveness, which is the value of the technique when deployed in the field. We need the fruit of effectiveness, not the seed of efficacy, to feed our patients. We also learned from the decades of experience with first-generation multicenter trials that simple protocols without economic induction can change guidelines, and cardiologists interested in SE are generous with their time and willing to do things that help them to work better and offer a contribution to clinically meaningful, patient-oriented research [19].

**Table 2:** Stress echo multicenter trials

Years	1990-2010	2016-2030
Study acronym	EPIC and EDIC	SE 2020
Main focus	CAD	CAD and beyond
Enrolling centers criteria	Selective	Inclusive
Stress	Dip and Dob	Exercise, dip (ado) and dob
Key parameters	RWMA	CFVR, B-lines, E/e’, etc.
Participating centers	10+	100+
Scientific societies role	Absent	Proactive

*CAD* coronary artery disease, *CFVR* coronary flow velocity reserve, *EDIC* echo-dobutamine international cooperative study, *EPIC*, echo-persantine international cooperative study, *RWMA* regional wall motion abnormalities

One generation later, in a totally different economic and scientific healthcare scenario, once again Italian and international cardiology creates a network to build up the missing evidence. At least in principle, SE2020 fully shares the four cornerstones of the landmark mega-trial GISSI, Gruppo Italiano Studio Streptochinasi nell’ Infarto, which can be summarized as follows [75]: 1) sponsorship by a respected, independent, not-for profit national society: it was the national Association of Hospital Cardiologists for GISSI, and now it is the Italian Society of Echocardiography for SE2020; 2) scientific coordination by a professional, public research institute: it was the Mario Negri of Milan for GISSI, it is now the National Research Council of Pisa for SE2020; 3) inclusivity, with the involvement of nearly all the interested professionals in the nation: it was over 300 coronary care units in GISSI, it is now > 100 echocardiography labs in SE2020; and 4) emphasis on clinically relevant topic and outcomes that directly impact every day patient care: it was thrombolytic therapy for GISSI, it is now be cardiac imaging in and outside CAD for SE2020. Hopefully, the results will help improve the practice standards for the Italian cardiology and echocardiographic community across the nation and the available practice-changing scientific evidence worldwide. In a rapidly evolving SE field, SE 2020 addresses the need for standardization clearly identified by the American Society of Echocardiography roadmap to 2020 in its research and technology recommendations [76]. The development of research registries is identified as a potentially important tool for assessing and improving the quality of care and a valuable platform for clinical research. Such registry data would be accessible to the research community, facilitating a broad range of clinical research on the effectiveness of echocardiography (in this case, of SE) for the improvement of patient management and outcome [76].

## Conclusion

The study officially starts in the very same days when American poet and singer Bob Dylan is awarded the Nobel prize for literature in December 2016 -- and perhaps also for SE "*the times they're in a-changin'* ". SE2020 will coordinate and channel the efforts of the echocardiographic community to keep SE where it stands now -- at center stage in guidelines of CAD and heart failure -- and to move on beyond CAD and above regional wall motion abnormalities, filling in the gaps in a currently evidence-poor field: "*The line it is drawn, the curse it is cast. The slow one now will later be fast, as the present now will later be past, the order is rapidly fadin'. And the first one now will later be last, for the times they are a changin'*".

## Abbreviations

CAD: Coronary artery disease
EDIC: Echo-dobutamine international cooperative study
EPIC: Echo-persantine international cooperative study
GISSI: Gruppo italiano studio streptochinasi nell’ infarto
SE: Stress echo
SIEC: Società italiana di ecocardiografia
